# Temporal and fertilizer-dependent dynamics of soil bacterial communities in buckwheat fields under long-term management

**DOI:** 10.1038/s41598-024-60655-w

**Published:** 2024-04-30

**Authors:** Susumu Morigasaki, Motomu Matsui, Iwao Ohtsu, Yuki Doi, Yusuke Kawano, Ryosuke Nakai, Wataru Iwasaki, Hisayoshi Hayashi, Naoki Takaya

**Affiliations:** 1 Faculty of Life and Environmental Sciences, University of Tsukuba, 1-1-1 Tennodai, Tsukuba, Ibaraki 305-8572 Japan; 2 Department of Biological Sciences, Graduate School of Science, University of Tokyo, 5-1-5 Kashiwanoha, Kashiwa, Chiba 277-0882 Japan; 3 Microbiology Research Center for Sustainability, University of Tsukuba, 1-1-1 Tennodai, Tsukuba, Ibaraki 305-8572 Japan; 4 Microbial Ecology and Technology Research Group, Bioproduction Research Institute, National Institute of Advanced Industrial Science and Technology, 2-17-2-1 Tsukisamu-Higashi, Toyohiraku, Sapporo, Hokkaido 062-8517 Japan; 5 Department of Integrated Biosciences, Graduate School of Frontier Sciences, University of Tokyo, 5-1-5 Kashiwanoha, Kashiwa, Chiba 277-0882 Japan; 6 Tsukuba-Plant Innovation Research Center, University of Tsukuba, 1-1-1 Tennodai, Tsukuba, Ibaraki 305-8577 Japan

**Keywords:** Ecology, Environmental sciences

## Abstract

This study integrated bacterial community and soil chemicals to characterize the soil ecosystem in an open upland field managed by six controlled fertilizer programs using the minimum amount of pesticides. Amplicon sequencing the 16S rRNA gene revealed that inorganic nitrogen fertilizer and compost altered the diversity and structure of the soil bacterial community throughout buckwheat (*Fagopyrum esculentum* Moench ‘Hitachiakisoba’) cultivation. The bacterial community comprised three clusters that contained bacteria that are prevalent in soils fertilized with nitrogen (cluster 1, 340 taxa), without nitrogen and compost (cluster 2, 234 taxa), and with compost-fertilized (cluster 3, 296 taxa). Cluster 2 contained more taxa in *Actinobacteriota* and less in *Acidobacteriota*, and cluster 3 contained more taxa in *Gemmatimonadota* compared with the other clusters. The most frequent taxa in cluster 1 were within the *Chloroflexi* phylum. The bacterial community structure correlated with soil chemical properties including pH, total organic carbon, SO_4_^2−^, soluble Ca^2+^. A co-occurrence network of bacterial taxa and chemicals identified key bacterial groups comprising the center of a community network that determined topology and dynamics of the network. Temporal dynamics of the bacterial community structure indicated that *Burkholderiales* were associated with buckwheat ripening, indicating plant-bacteria interaction in the ecosystem.

## Introduction

Biogeochemical activities of soil microbes decompose organic matter and contribute to carbon and nitrogen cycles in ecosystems^1,2^, and in anthropogenically-established agricultural fields. Farmer-friendly inorganic fertilizers have been supplying missing nutrients and increased crop productivity at low cost without being labor-intensive for over a century^3^. However, the intensive or long-term application of the inorganic fertilizers disturbs microbial communication in soil ecosystems, and adversely affects crop yield and quality^4,5^. Although no technology is yet available to isolate and characterize all the soil microorganisms involved in these phenomena, analysis of bacterial communities based on sequencing 16S ribosomal RNA (rRNA) genes in soil ecosystems has identified numerous uncultivated microbes^6^ and revealed a global diversity of soil microbial communities^7^. Therefore, a better understanding the structure and function of the soil microbial community and its relationships with crops and soil nutrients is required to develop sustainable agricultural practices.

Agricultural fields that have been systematically managed over the long term are stable ecosystems that can serve as models, because interactions among a vigorous microbial community, soils, and crops should be reproducible^8,9^. The purpose of this study was to determine the temporal and fertilizer-dependent dynamics of soil bacterial communities in buckwheat fields under long-term management. In our target field, systematic management over three decades with minimum pesticides has resulted in stable crop production, indicating stable control of the bacterial community structure and soil properties. The open upland ecosystem is another unique feature of the test field and a promising model that fills the gap between actual farmland and systematic bacterial community studies in laboratories and/or greenhouses. Therefore, analysis of these test fields should reveal the dynamics of soil microbial communities in upland agricultural fields that depend on fertilization protocols and crop growing periods.

This study investigated six test plots under different fertilization conditions in a test field during a single season of buckwheat (*Fagopyrum esculentum*) cultivation. Buckwheat is a popular crop from which edible seed flour is derived. We analyzed extensive datasets of bacterial community structures, soil chemical properties, and crop phenotypic traits indicative of changes caused by fertilizer programs and cultivation stages in a test field. Network analyses indicated that pH, total organic carbon (TOC) and SO_4_^2−^ are core factors of the bacterial community structure. The network comprises a center and three groups, in which distinct bacteria maintain specific topologies and dynamics.

## Results

### Soil and crop data of six plots in fertilizer test field

**Figure 1:**
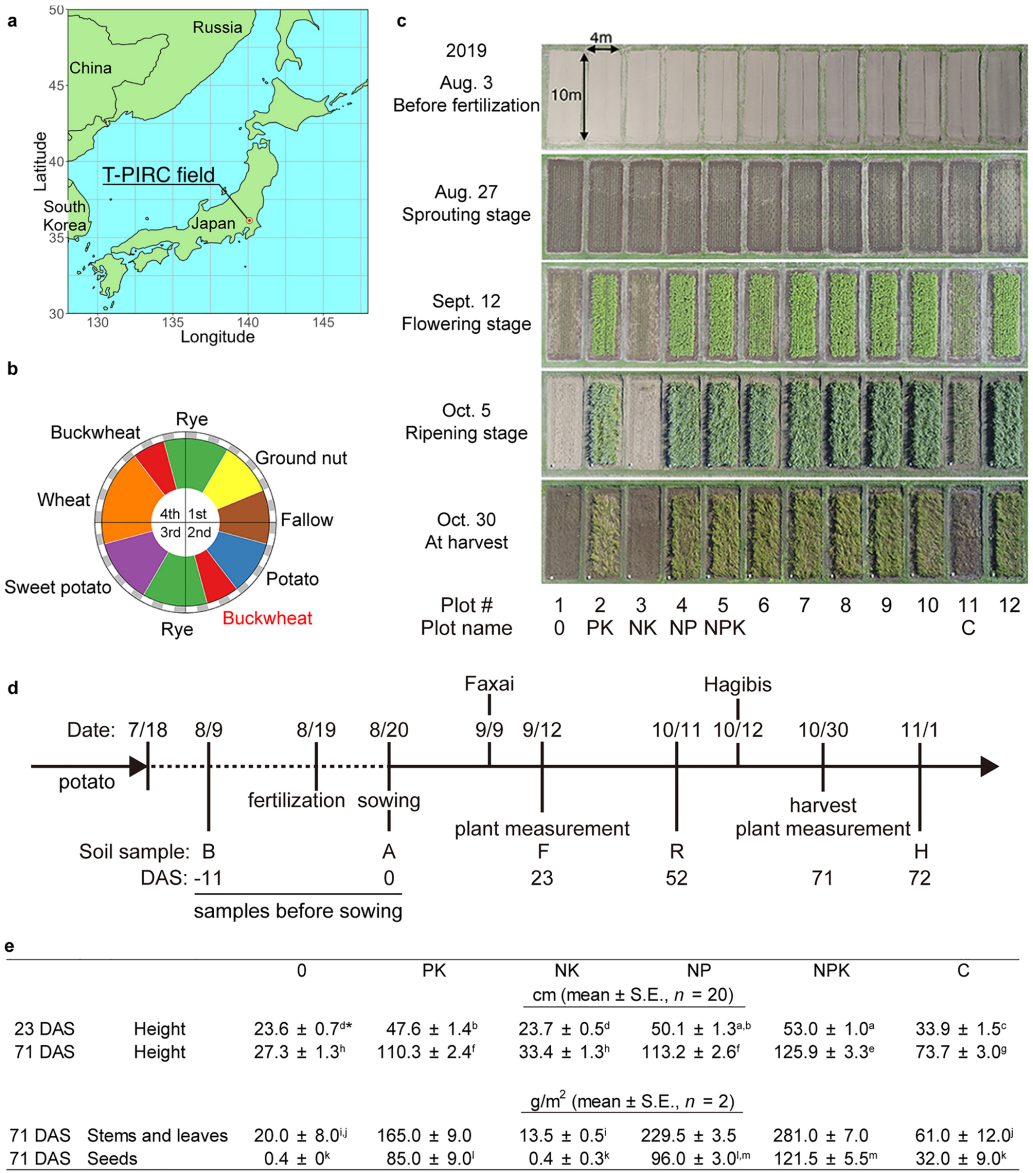
Fertilizer test field and experimental design. (**a**) Location of T-PIRC in Tsukuba, Japan. (**b**) Four-year rotation cycle of crop cultivation managed since 1986. Buckwheat cultivation season is colored red. (**c**) Aerial view of fertilizer test field during buckwheat cultivation. Effects of fertilizers N, nitrogen; P, phosphate; K, potassium; and C, compost were assessed in plots 1–5 and 11. (**d**) Buckwheat cultivation and experiment schedule. Dashed line indicates interval between potato harvesting and buckwheat cultivation. Buckwheat seeds were sown on 8/20 in 2019. Soil was sampled before fertilization (B, 8/9), after fertilization (pre-sowing, A, 8/20), flowering (F, 9/12), ripening (R, 10/11), and post-harvest (H, 11/1). Plant traits were measured on 9/12 and 10/30. Faxai and Hagibis are typhoons that attacked the area. (**e**) Above-ground height of buckwheat on 9/12 and 10/30 and yields of harvested stems, leaves, and seeds. *Values with same letters do not significantly differ (*p* ≥ 0.05, Tukey–Kramer test). *DAS* days after sowing, *0 DAS* day of sowing, *−11 DAS* 11 days before sowing.

We targeted a fertilizer test field that comprised one plot without fertilizer (plot 0), and one each fertilized with phosphate (P) and potassium (K; plot PK), nitrogen (N) and potassium (plot NK), nitrogen and phosphate (plot NP), three macronutrients (plot NPK), and compost (plot C) (Fig. 1a–c)^10^. Soils in the plots were collected throughout buckwheat cultivation before (B) and after (A) fertilization, flowering (F), ripening (R), and post-harvest (H) (Fig. 1d). Supplementary Table S1 includes the data along with information about the applied fertilizers and cultivation stages. Weather parameters, such as temperature, rainfall, humidity, and solar radiation were monitored in real time (Supplementary Fig. S2).

The averaged coefficients of variation (CV) for chemical component and bacterial abundance were 0.26 and 0.59, respectively (Supplementary Fig. S1). This reflected the homogeneity of the soil in each plot, stable soil properties during long-term agricultural management, and the reproducibility of the results. Chemical fertilizers increased crop growth estimated as stem height, plant biomass and seed yield (Fig. 1e). Poor plant growth in plot NK could be explained by the limited availability of phosphate for buckwheat in an andosol that adsorbs > 23 g phosphate/kg^10^. Compost had little effect on crop growth, suggesting its role as a soil conditioner rather than a fertilizer.

### Cultivation and fertilization alter chemical components of soils

**Figure 2:**
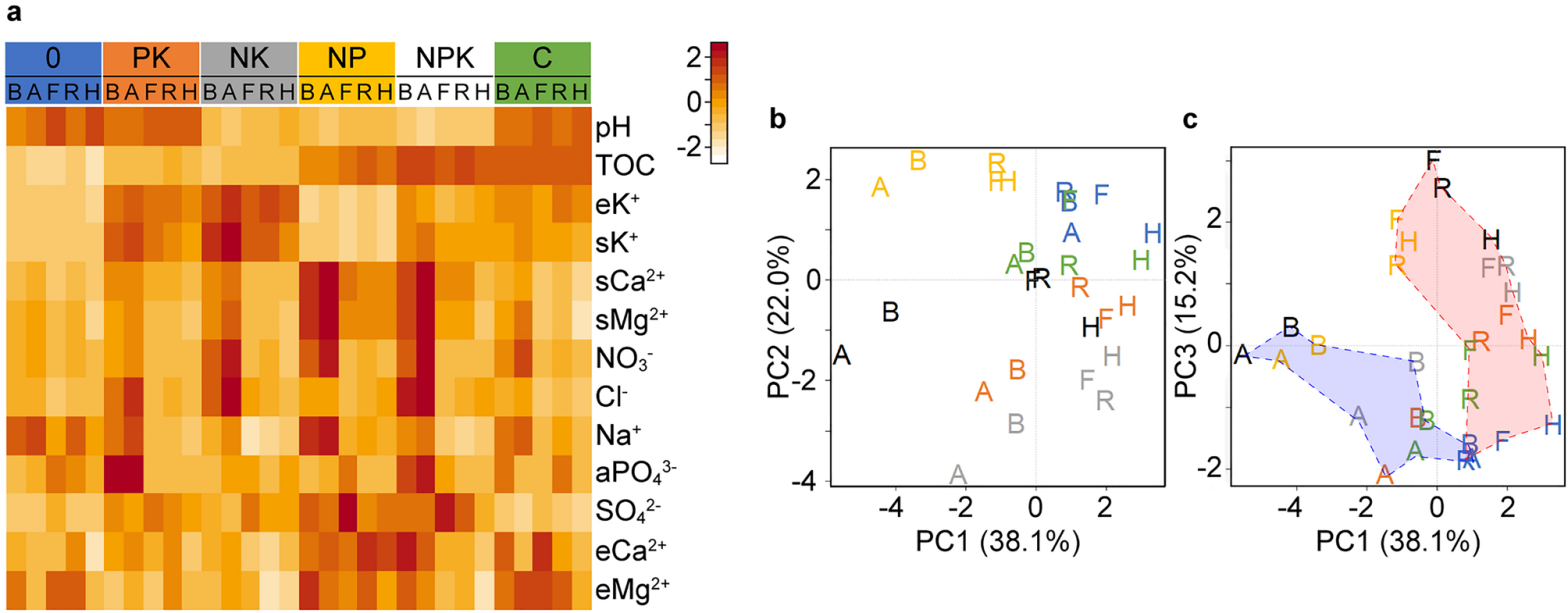
Soil chemical components that correlated with cultivation and fertilization. (**a**) Heatmap of soil chemical components. Median values of chemical components obtained from four batches of soil samples per plot and cultivation stage were standardized to create Supplementary Dataset 2 online, and processed using heatmap.2 in R. Soil was sampled before (B) and after (A) fertilization, flowering (F), ripening (R) and harvest (H). (**b**,**c**) Scatter plots obtained by PCA of chemical components in Supplementary Dataset 2. Proportions of variance (%) of principle components are shown in parentheses on axis labels. Plot color codes: 0 (blue), PK (orange), NK (grey), NP (yellow), NPK (black), and C (green). Blue and red areas in panel C grouped soils sampled before (B and A) and after (F, R, and H) sowing, respectively (*p* = 0.001, PERMANOVA).

We systemically monitored the following chemical components of soils at the cultivation stages; total organic carbon (TOC), NO_3_^−^, NH_4_^+^, Na^+^, Cl^−^, SO_4_^2−^, soluble (s) K^+^, sCa^2+^, and sMg^2+^ and exchangeable (e) K^+^, eCa^2+^, eMg^2+^, and available (a) PO_4_^3−^, and pH (Fig. 2a and Supplementary Fig. S3). Concentrations of nitrate nitrogen (NO_3_–N) in the NK, NP, and NPK plots were high (*p* < 0.003) in stage A soon after fertilization. These values decreased during stages F, R, and H accompanied by crop growth (*p* < 0.05). The concentrations of NH_4_^+^ were below the limits of detection (< 0.07 mg/kg dry soil, CV = 1.5) in soils from the six test plots (Fig. 2a and Supplementary Figs. S1 and S3), indicating rapid nitrification and nutritionalization of the NH_4_^+^. The nitrogen fertilizer also supplied its counter anion SO_4_^2−^ to the soils. However, the SO_4_^2−^ level did not increase after fertilization (*p* > 0.12) but increased 1.5- to 1.9-fold (*p* < 0.05) at the flowering stage (24 days after fertilization). The pH was lower in soil from plots NK, NP, and NPK than in the other plots (5.4 ± 0.1 vs. 6.0 ± 0.1, *p* < 0.0001), which agrees with the fact that inorganic nitrogen fertilizers acidify many types of soils^11^. Levels of eK^+^ were higher in soils from plots PK, NK, NPK and C than plots 0 and NP (~ 35 vs. ~ 3 mg/kg dry soil, *p* < 0.05). Most soil samples contained < 10 mg/kg of aPO_4_^3−^ dry soil (CV = 1.4) which was below the limits of quantitation (Supplementary Figs. S1 and S3). Adding phosphate did not affect the soil aPO_4_^3−^ level much. More TOC was found in the NP and NPK plots and C soils than in the other plots (30 ± 1 vs. 23 ± 1 g/kg dry soil, *p* < 0.001). We compared soil chemical properties using principal component analysis (PCA) (Fig. 2b,c). Soils in the PC1‒PC3 scatter plot differed between before and after fertilization (Fig. 2c; B and A in blue area), compared with the flowering, ripening and after-harvest stages (Fig. 2c; F, R, and H in red area) (*p* = 0.001). These results suggested that crop growth altered soil chemical properties that manifested mostly as decreased levels of soluble Na^+^, sK^+^, sCa^2+^, sMg^2+^, NO_3_^−^, and Cl^−^ ions (Fig. 2a and Supplementary Fig. S3).

### Fertilizer-linked bacterial community structures throughout all crop cultivation stages

**Figure 3:**
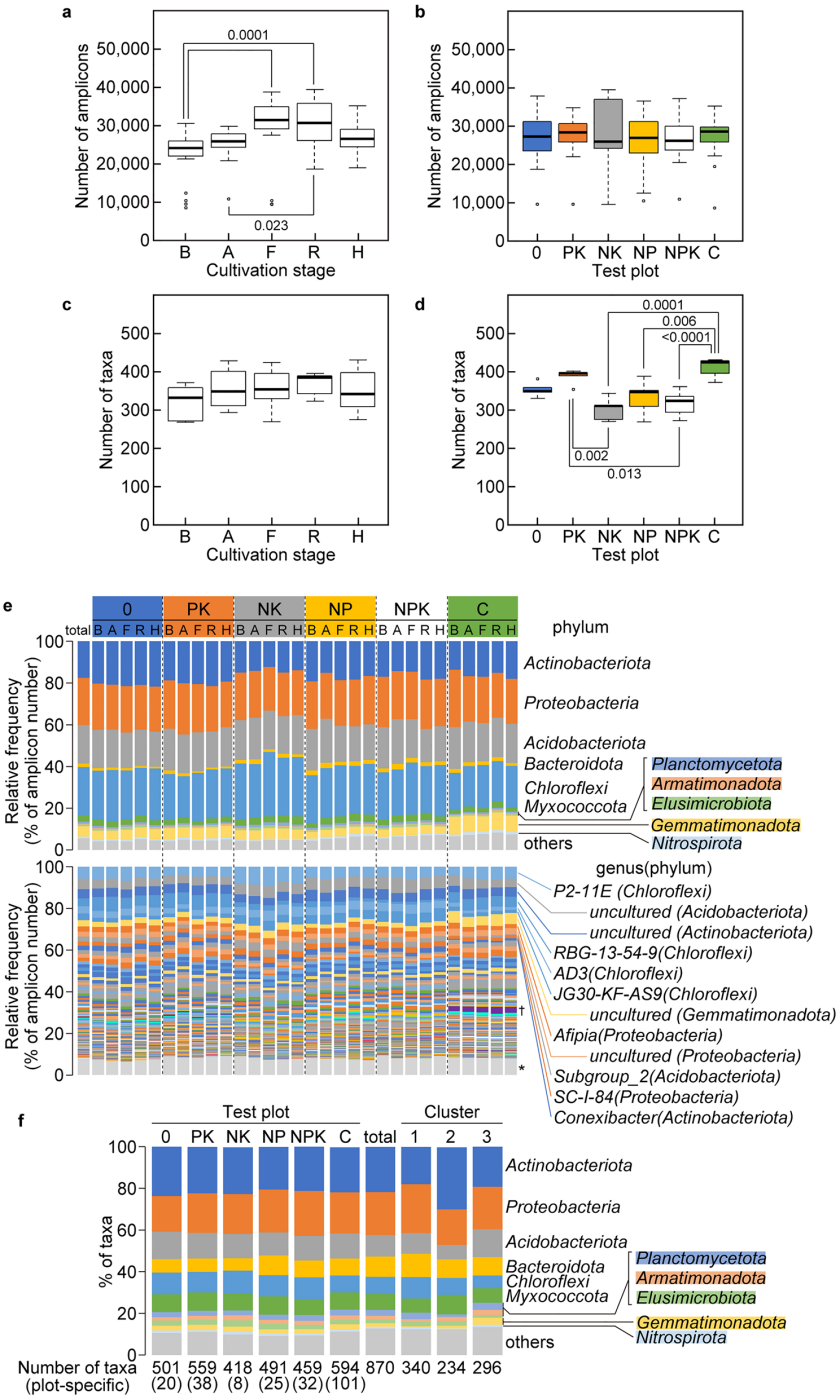
Bacterial community structures in test plots. (**a–d**) Box plots summarize 16S rRNA gene amplicon sequencing. Sequenced amplicons in Supplementary Table S1 were counted for each cultivation stage (**a**) and test plot (**b**). Taxa at species level in Supplementary Dataset 1 were counted for each cultivation stage (**c**) and test plot (**d**). Results were visualized using boxplot function in R. Thick line in box, median; top of box, third quartile (Q3); bottom of box, first quartile (Q1); upper whisker, maximum value (< Q3 + 1.5 × [Q3 − Q1]); lower whisker, minimum value (> Q1 − 1.5 × [Q3 − Q1]). Unfilled circles indicate outliers. Thin lines indicate significant differences (*p*-values determined by Tukey–Kramer test). (**e**) Bar plots show relative frequencies per phylum (top) or per genus (bottom) that were calculated using Supplementary Dataset 1. Genus names of 12 frequent genera were described with phylum names in parentheses. *Taxa with no description of genus. ^†^*Latescibacteraceae* (*Latescibacterota*). (**f**) Bar plots show diversity of test plots and taxon clusters (see Fig. 4h). The numbers of taxa per phylum were counted using Supplementary Dataset 1. The total numbers of taxa in test plots are shown at bottom of bars with the numbers of plot-specific taxa in parentheses. Abbreviations of cultivation stages: before (B) and after (A) fertilization, flowering (F), ripening (R) and harvest (H).

We sequenced 3,222,131 (26,851 ± 6974/sample) gene amplicons and Qiime 2 analysis identified 870 taxonomic units (taxa) that were grouped into 33 phyla, 86 classes, 150 orders, 207 families, and 274 genera (Fig. 3a–d). *Actinobacteriota*, *Proteobacteria*, *Acidobacteriota*, *Bacteroidota*, *Chloroflexi*, and *Myxococcota* were the most diverse phyla in the plots, accounting for 86% and 78% of the total number of sequenced amplicons (Fig. 3e top) and identified taxa (Fig. 3f), respectively. Nineteen genera were distributed in the six test plots at a high frequency (z-score > 1.96) and corresponded to 50% of the total number of sequenced amplicons (Fig. 3e bottom, Supplementary Table S2). These include 15 genera identified metagenome analyses and without physiological characterization, and four genera characterized as *Afipia*, *Conexibacter*, *Sphingobium*, and *Gaiella*. These genera occupied the test plots at different ratio (p < 0.05, Tukey–Kramer test), indicating that the fertilizer program differentiates taxa at genus level (Supplementary Table S2). The frequency of these genera did not significantly differ among the cultivation stages except for one uncultured genus of the phylum *Proteobacteria*, which differed between cultivation stages A and F (p < 0.05) (Supplementary Table S2). The numbers of taxa did not differ among the cultivation stages (Fig. 3c). The Shannon index (H′) was larger for the six test plots than the individual plots (5.05 vs. 4.54–4.96), implying different bacterial community structures among them (Table 1). The H′ was larger in soil from plots PK and C (4.88 ± 0.04 and 4.93 ± 0.02) than soils from plots 0, NK, and NPK (4.75 ± 0.06, 4.66 ± 0.07, and 4.77 ± 0.03; *p* < 0.002, Tukey–Kramer test). These results indicated a more diverse bacterial community structure in plot C, which was supported by finding more taxa in soil from this plot than those supplemented with nitrogen fertilizer over the long term (Fig. 3d). The prevalence of bacteria in the phylum *Gemmatimonadota* was also higher in soil from plot C (*n* = 5; *p* < 1 × 10^–7^, Tukey–Kramer test) (Fig. 3e; yellow), which also contained far more specific taxa (*n* = 101) than the other plots (Fig. 3f).

**Table 1:** Alpha-diversity of soil bacteria in test plots. Bacterial community data were rarefied in coverage-based manner using rareslope, unlist, lapply and rrarefy functions in R. Shannon index (H′) was determined using the diversity function. ^a^H′ was tested by Tukey–Kramer test using the TukeyHSD function (*n* = 5). ^b^Values with the same letter are not significantly different (*p* > 0.05). ^c^Alpha-diversity of 6 tested plots is shown as H′ for all rarefied bacterial community data.

Plot	Stage	H'	Mean	SD	TK^a^
0	B	4.77	4.75	0.06	a, c^b^
	A	4.72			
	F	4.66			
	R	4.83			
	H	4.75			
PK	B	4.83	4.88	0.04	b
	A	4.93			
	F	4.86			
	R	4.92			
	H	4.88			
NK	B	4.71	4.66	0.07	a
	A	4.68			
	F	4.54			
	R	4.73			
	H	4.65			
NP	B	4.72	4.84	0.09	b, c
	A	4.84			
	F	4.86			
	R	4.97			
	H	4.82			
NPK	B	4.75	4.77	0.03	a, c
	A	4.72			
	F	4.77			
	R	4.80			
	H	4.79			
C	B	4.90	4.93	0.02	b
	A	4.93			
	F	4.95			
	R	4.93			
	H	4.96			
6 test plots^c^		5.05			

**Figure 4:**
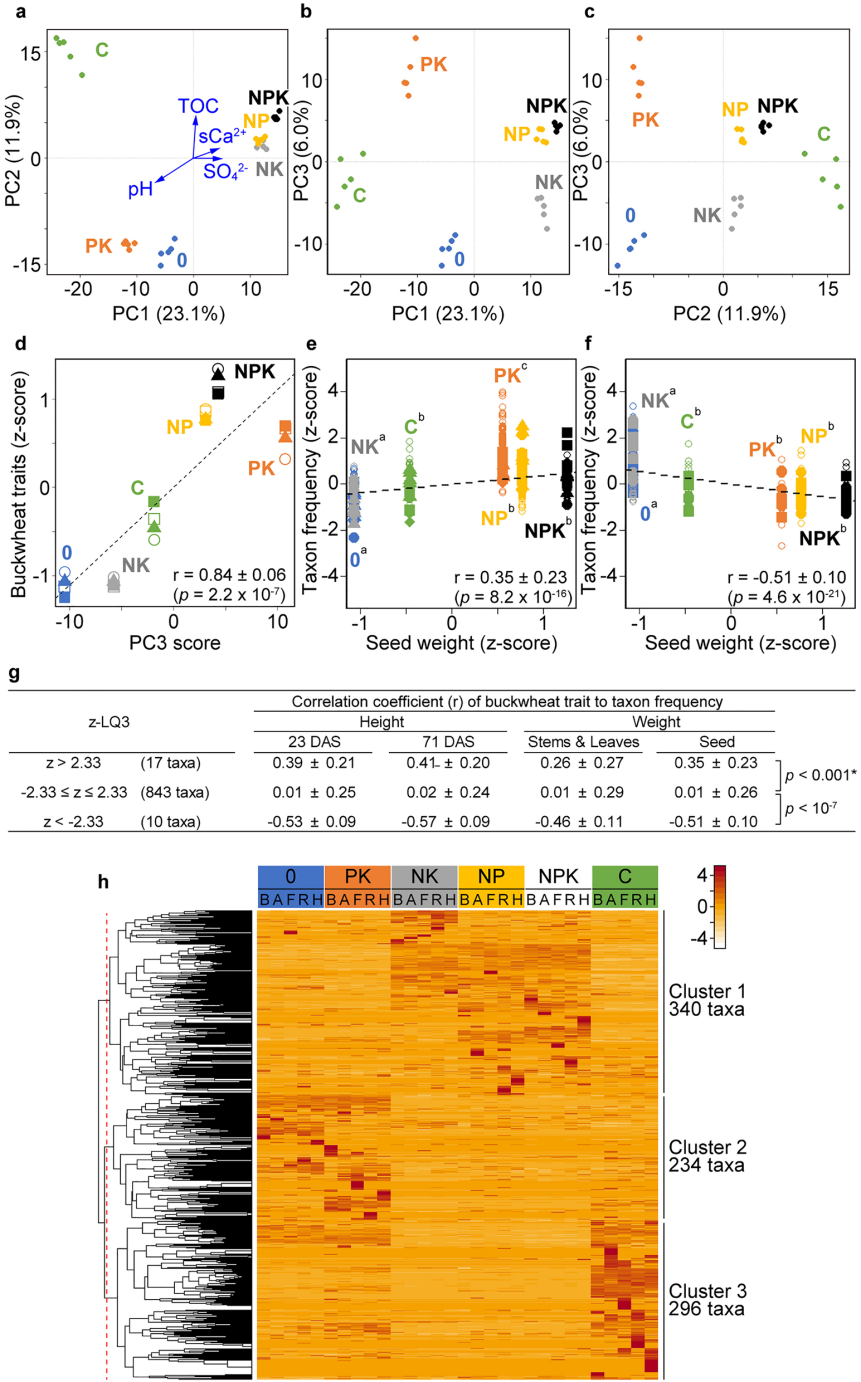
Bacterial community structures associated with soil chemical properties and crop traits. (**a‒c**) Scatter plots show PCA of soil bacterial community. Bacterial community data in Supplementary Dataset 2 were analyzed using prcomp in R. Plots 0 (blue), PK (orange), NK (grey), NP (yellow), NPK (black), and C (green). Five dots in each plot correspond to five cultivation stages. Parentheses on axis labels show proportions of variances. Significance in PC1 and PC2 scores among the three groups was analyzed using Tukey–Kramer test (*p* < 10^–4^ and* p* < 10^–6^, respectively). (**a**) PC1-PC2. Ordination vectors of pH, TOC, sCa^2+^, and SO_4_^2−^ (blue arrows, *p* < 0.01) projected using envfit and standardized chemical component data in Supplementary Dataset 2. (**b**) PC1–PC3. (**c**) PC2–PC3. (**d**) Correlations between PC3 scores and agronomically important buckwheat traits, namely height at 23 (unfilled squares) and 71 (filled squares) DAS, yield of stems and leaves (unfilled circles), and seed yield (filled triangles). (**e**,**f**) Correlations between bacterial taxa and seed weight. Taxa that positively contributed to PC3 scores were evaluated according to load quantity scores (z-LQ3) and their correlations with seed weight were analyzed. Test plots with same letters do not significantly differ (*p* ≥ 0.05, Tukey–Kramer test). (**e**) Taxa with positive contributions to PC3 scores (z-LQ3 > 2.33) and seed weight. Filled symbols, taxa with high *r* values; squares, *Rhodococcus* of *Actinobacteriota*; circles, *CCD24* of *Proteobacteria*; triangles, *Gaiellales* of *Actinobacteriota*; diamonds, *Diplorickettsiaceae* of *Proteobacteria*. Unfilled circles indicate 13 other taxa. (**f**) Taxa that negatively contributed to PC3 scores (z-LQ3 <  − 2.33) and seed weights. Filled symbols, taxa with lowest *r* values; Squares, *mle1-27* of *Myxococcota*; circles, *P2-11E* of *Chloroflexi*. Unfilled circles indicate other eight taxa. (**g**) Correlations between bacterial taxa and buckwheat traits. Taxa were grouped according to z-LQ3 (2.33 cutoff) and correlations with buckwheat traits were statistically analyzed using *t-test (“Materials and methods”). (**h**) Heatmap of taxon frequency and hierarchical clustering generated from Supplementary Dataset 2 using heatmap.2 and hclust functions in R that identified clusters 1, 2 and 3. Abbreviations of stages: before (B) and after (A) fertilization, flowering (F), ripening (R) and harvest (H).

Similarity among the bacterial community structures in plot soils evaluated by PCA of bacterial abundance revealed a relationship between soil bacterial community structures and fertilizers (Fig. 4a‒c). The PC1-PC2 scatter plot classified soils into groups with added compost, nitrogen fertilizer, or neither (*p* < 10^–4^ (PC1), *p* < 10^–6^ (PC2), Tukey–Kramer test) (Fig. 4a). The third component (PC3) phosphate fertilization-dependently discriminated the plots (Fig. 4b). Soils containing abundant TOC (NP, NPK, and C) were segregated from the other soils in the PC2–PC3 scatter plot (Fig. 4c). The PC scores for the respective test plots at the various cultivation stages were similar. The fertilizer program established plot-specific bacterial community structures that were significantly conserved throughout the cultivation stages.

### Bacterial community structures are associated with chemical components in soils

The PCA results of the 16S rRNA amplicons classified soils into 3 groups, and thus bacterial taxa were hierarchically clustered and sorted into three clusters (Fig. 4a,h and Supplementary Table S3). Cluster 1 comprised taxa that were prevalent in the NK, NP and NPK plots fertilized with nitrogen (Fig. 4h), whereas phylum diversity was similar between cluster 1 and the total profile (Fig. 3f). Bacteria in cluster 2 were prevalent in plots 0 and PK (Fig. 4h). The number of taxa belonging to the phylum *Actinobacteriota* accounted for 30% of cluster 2 compared with 18% and 19% of clusters 1 and 3 (Fig. 3f), and no taxa belonging to the phylum *Chloroflexi* were prevalent in cluster 2 (Supplementary Table S4). Cluster 3 comprised bacteria that were prevalent in plot C (Fig. 4h). The phylum *Gemmatimonadota* was more diverse in cluster 3 (10 taxa) than in clusters 1 and 2 with two and three taxa, respectively (Fig. 3f) and more frequent in plot C (Fig. 3e, top). The most frequent taxa in the phylum *Gemmatimonadota* belonged to cluster 3 (Supplementary Table S4). Cluster 3 did not contain a frequent taxon belonging to the phylum *Actinobacteriota* (Supplementary Table S4).

We investigated correlations between chemical components and bacterial community structures that have remained debatable^12^. The profiles of pH, SO_4_^2−^, TOC and sCa^2+^ in soils under fertilization management at the cultivation stages correlated with those of 175, 49, 42, and 37 taxa, respectively, with some overlap (|*r*|> 0.6) (Supplementary Fig. S4 and Supplementary Table S3). Ordination analysis projected vectors on the PC1–PC2 scatter plot (Fig. 4a) to elucidate correlations between the chemical components and bacterial community structures. The coefficients of determination (*r*^2^) that reflect vector lengths for pH, TOC, SO_4_^2−^, and sCa^2+^ were 0.94, 0.67, 0.38, and 0.32, respectively (*p* < 0.01). The results indicated that pH, TOC, SO_4_^2−^ and sCa^2+^ correlate with, and are predictors of the bacterial community structure in soil.

### Bacterial correlations with crop phenotypic traits

The correlation coefficients of seed weight among components of clusters 1, 2, and 3 were 0.25 (*p* = 1.30 × 10^–30^), − 11 (*p* = 2.81 × 10^–6^), and − 0.10 (*p* = 1.81 × 10^–4^), respectively, indicating that cluster 1 weakly correlates, whereas clusters 2 and 3 do not correlate with seed weight. The PC3 score in the PCA of bacterial abundance were higher for the phosphate-fertilized PK, NP, and NPK, than the other test plots (Fig. 4b). Considering that phosphate fertilization increased the buckwheat biomass (Fig. 1e), we compared PC3 scores and the buckwheat phenotypic traits. The results showed that they closely correlated (*r* = 0.84 ± 0.06, *p* = 2.2 × 10^–7^) (Fig. 4d). The load quantity (LQ) for the PC3 score (LQ3) evaluated bacterial taxa that contributed to PC3 scores. Taxa that contributed the most to the PC3 score (|z-LQ3|> 2.33), accounting for 10% of the cumulative contribution score contained 17 positive contributors among which were the four taxa, *Rhodococcus* and *Gaiellales* of the phylum *Actinobacteriota*, *CCD24*, and the *Diplorickettsiaceae* of the phylum *Proteobacteria* (Supplementary Table S5). The taxa also contained 10 negative contributors including the two taxa, *mle1-27* and *P2-11E* of *Myxococcota* and *Chloroflexi* phyla, respectively. The frequencies of these six taxa correlated with buckwheat seed yields (|*r*|> 0.6) (Fig. 4e,f; filled symbols; Supplementary Table S5). The 27 taxa also correlated with biomass (height and weight) with a slightly weak correlation in the latter (Supplementary Fig. S5), while the extent of their correlation was apparently higher than the remaining 843 taxa (*p* < 0.01 and *p* < 10^–7^ for positive and negative contributors, respectively (Fig. 4g). These results indicated that the 27 taxa, especially the six taxa that correlate with buckwheat traits (|*r*|> 0.6, bold in Supplementary Table S5), are associated with maintaining the ecosystem in phosphate-fertilized fields and increasing buckwheat biomass.

### Pivotal components in network topology determined by co-occurrence analysis

**Figure 5:**
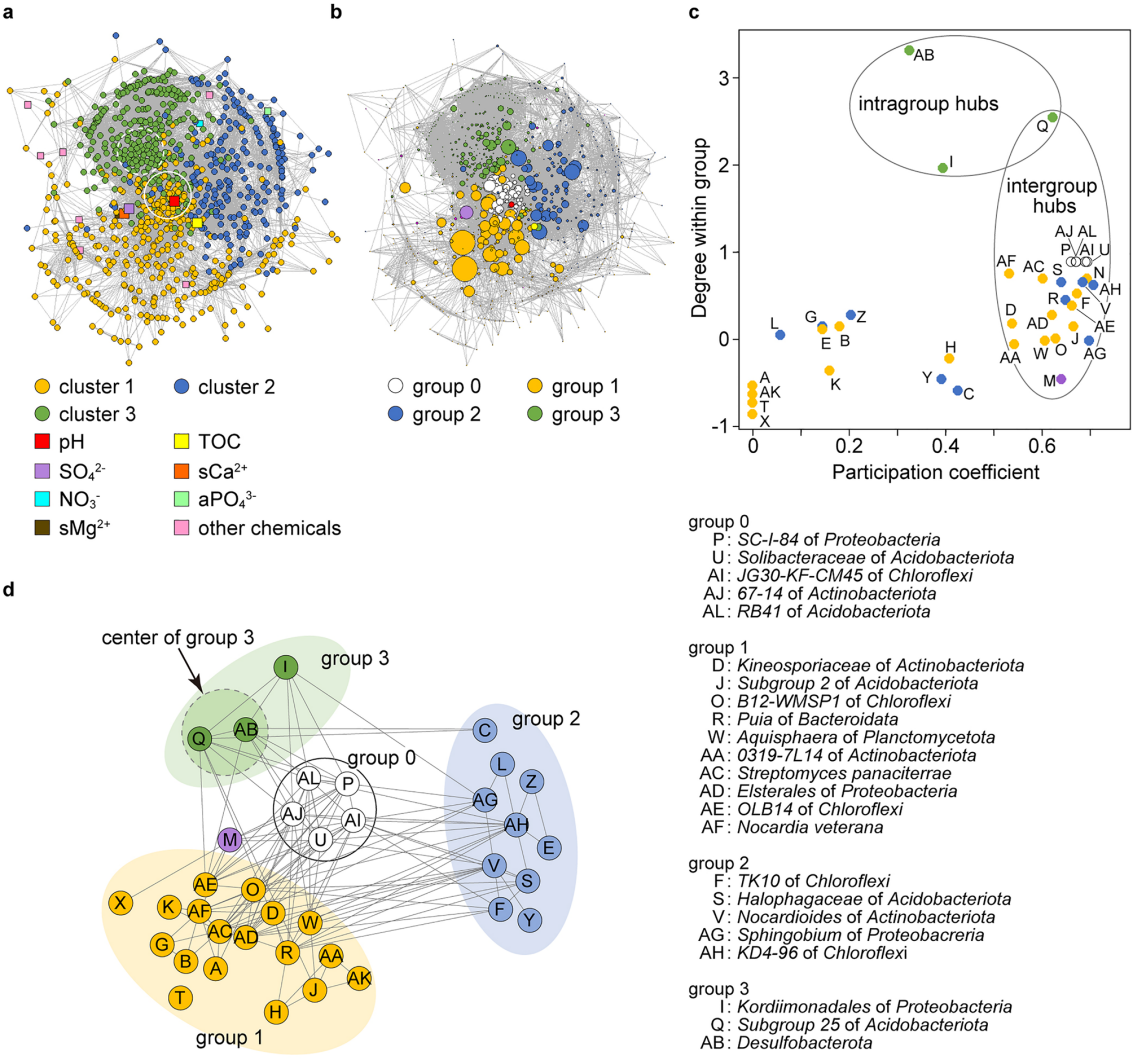
Pivotal components in network topology determined by co-occurrence analysis. (**a**) Co-occurrence network of bacterial community and chemical components using Supplementary Dataset 2 and the cor functions in R. Nodes are color-coded by bacterial clusters 1 (ocher), 2 (blue), and 3 (green). Square nodes indicate chemical components. White filled circles surround centers in network (solid line) and cluster 3 (dashed circle) (Materials and Methods). (**b**) Betweenness centrality in network. Node size is proportional to betweenness centrality determined using degree and betweenness functions in R. Node groups 0, 1, 2 and 3 are colored white, ocher, blue, and green, respectively. Chemical components are colored as shown in panel A. (**c**) Participation coefficient and within-group degrees of nodes identified 25 intergroup and intragroup hubs as described in Guimerà et al.^14^. Node group and chemical components are colored as described in panel (**b**). (**d**) Schema of network with classified hubs. Edges connect nodes with close correlations (|*r*|> 0.6). Right, annotated hubs (Supplementary Table S6 shows all annotations), Dashed circle, center of group 3.

Co-occurrence among bacteria and chemical components in soils were visualized as network diagrams (Fig. 5). The three bacterial clusters (Fig. 4h) were also separated within the network (Fig. 5a). In addition to most chemical components at the network periphery, pH, TOC, SO_4_^2−^, sCa^2+^, and sMg^2+^ were located inside the network, indicating strong links with the bacterial community structure in soils from the six test plots. These findings agreed with the ordination results (Fig. 4a), and further indicated a correlation between sMg^2+^ and the soil bacterial community structure.

The topological analysis identified two complete graphs that contained over 45 nodes (> 5% of total nodes) in the network. The larger of the two complete graphs show pH (red square) and 58 taxa in clusters 1 and 3 (Fig. 5a; solid circles). This group conceivably represents the center of the network, and thus is hereinafter referred to as group 0. Groups 1 and 3 were defined by eliminating the components of group 0 from clusters 1‒3, and group 2 comprised components that perfectly overlapped cluster 2. Another complete graph comprised 56 taxa exclusively in group 3 and were thus considered to comprise the center of group 3 (Fig. 5a; dashed circle). The nested network topology of group 3 was responsible for a unique community structure established by composting, which strongly affects soil bacteria.

**Figure 6:**
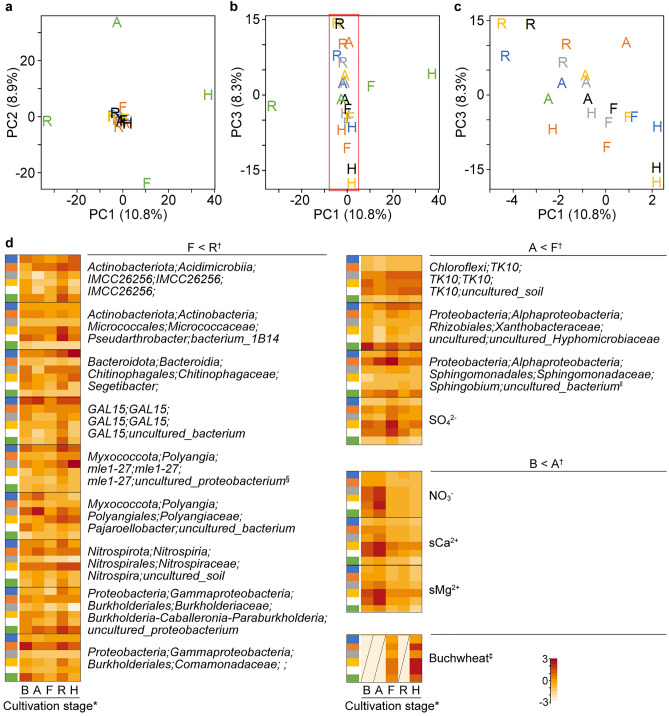
Soil bacteria associated with buckwheat cultivation. (**a**‒**c**) PC1–PC2 and PC1–PC3 scatter plots of difference in frequency before and at each cultivation stage (Δf). A, Δf(A) = f(A) − f(B); F, Δf(F) = f(F) − f(A); R, Δf(R) = f(R) − f(F); H, Δf(H) = f(H) − f(R). Principle components were analyzed and taxon frequency was calculated using Supplementary Dataset 1 and prcomp in R. Proportions of variance (%) are shown in parentheses. Plot color codes: 0 (blue), PK (orange), NK (grey), NP (yellow), NPK (black), and C (green). Panel (**c**) is magnification of red rectangle in panel (**b**). Significance between stage R and other stages tested by PERMANOVA (*p* < 0.002). (**d**) Heatmap of bacterial taxa and chemical components prepared using heatmap.2 and hclust functions in R and Supplementary Dataset 2 after standardization. Colored bars on left indicate test plots as described in panels (**a**–**c**). *Abbreviations of stages: before (B) and after (A) fertilization, flowering (F), ripening (R) and harvest (H). ^†^*p* < 0.05 (*n* = 24, *t*-test). ^‡^Above-ground height of buckwheat (*p* < 0.05, F vs. H; *t*-test). ^§^Taxon *mle1-27* of *Myxococcota* phylum contributed to PC3 (Supplementary Table S5). ^ǁ^*Sphingobium* of *Proteobacteria* phylum is a hub in co-occurrence network (AG in Fig. 5d and Supplementary Table S6).

Topological property parameters of the network were summarized (6 test plots in Supplementary Fig. S6). Betweenness centrality represents the degree to which a given node falls on the shortest paths toward other nodes. It is used to evaluate the potential for a node to control a network structure as hubs^13–15^. This study focused on 38 nodes with high betweenness centrality (z-scores > 2.24) to define network hubs (Supplementary Table S6). The network in Fig. 5b was recreated so that node size was proportional to the betweenness centrality values and color-coded by their involved groups. Furthermore, standardized within-group degrees and participation coefficients (degrees of contribution to intergroup linkage) classified 25 nodes into intergroup and intragroup hubs (Fig. 5c and Supplementary Table S6)^15,16^. The results are shown as a model schema (Fig. 5d), indicating that five nodes in group 0 are intergroup hubs that link group 0 to groups 1 and 3. Groups 1 and 2 are linked to group 0 by 10 (D, J, O, R, W, AA, AC, AD, AE and AF) and five (F, S, V, AG, and AH) intergroup hubs in the respective groups (Supplementary Table S6). The *Nocardioides* of *Actinobacteriota* (V) and the uncultured *OLB14* of *Chloroflexi* (AE) had additional intergroup linkages between groups 2 and 1 and between groups 1 and 3, respectively. A few hub linkages and a few overall edges indicated a looser correlation between groups 2 and 3. The uncultured *Desulfobacterota* (AB) and the proteobacterial *Kordiimonadales* (I) resided in group 3 and were intragroup hubs. The *Subgroup 25* of the *Acidobacteriota* (Q) was an intragroup hub of group 3 and also functioned an intergroup hub between groups 0 and 3. Hub Q residing in the center of group 3 played an important role in maintaining the structures of this group and the entire network through an intergroup connection. *Kitasatospora_sp.* of the phylum *Actinobacteriota* (L) and *uncultured_Desulfuromonadale* of the phylum RCP2-54 (Z) were also PC3-contributors and belonged to group 2 (Supplementary Tables S6 and S7). The frequency of *Sphingobium* of the phylum *Proteobacteria* (AG) in group 2 increased at the flowering stage (Fig. 6d). In addition, SO_4_^2−^ (M) was the sole hub of the soil chemical components and mediated linkages between groups 0 and 1, which concurs with its close relationship with the bacterial community structure (Fig. 4a). Sulfate is a key chemical component in the establishment of a bacterial community network.

Co-occurrences of taxa and chemicals in respective test plots and cultivation stages were shown in Supplementary Fig. S6. Degree and closeness centralities were significantly different among test plots except for degree centrality between plots 0 and NP. Betweenness centrality of plots PK and C was higher than those of the other plots. Average path length of plot C was significantly higher than that of plot NK. None of the centralities and average path lengths differed among the cultivation stages except for degree centrality of stage B, closeness centrality between stages A and F and stages F and R, and average path lengths between stages F and R and stages R and H. Differences in the topological parameters were larger among the plots than among the cultivation stages.

### Soil bacteria associated with buckwheat growth

We investigated bacterial responses to crop cultivation by calculating differences in bacterial frequency between before and at defined cultivation stages (Δf) (Supplementary Fig. S7). The PCA scatter plots of Δf indicated similar temporal changes in soil profiles among the test plots except plot C, where the bacterial community structure obviously changed during cultivation stage transitions (Fig. 6a,b). These findings agreed with the notion that compost established a unique bacterial community structure (Fig. 4). The PC1–PC3 scatter plot of Δf distinguished R (Fig. 6c) from other stages, indicating specific changes in the soil bacterial community structures as buckwheat seeds ripen (*p* < 0.002, PERMANOVA). The Δf values of nine taxa were positive at the ripening stage, Δf(R) in the six test plots, and this was probably associated with the transition from the F stage (Fig. 6d; “F < R”). Seven of the nine taxa belonged to the phyla *Actinobacteriota*, *Bacteroidota*, *Myxococcota*, and *Proteobacteria*, which dominated in the six test plots (Fig. 3f). The others were affiliated with the phylum *Nitrospirota* and the *Candidatus* phylum *Ca. GAL15*, which includes rarely cultivated bacterial lineages. Two of the proteobacteria belonged to the order *Burkholderiales*, which agreed with their enrichment in the rhizosphere^17^. Three taxa were frequent at the flowering stage: the *Candidatus* class *TK10* of the phylum *Chloroflexi* and two of the class *Alphaproteobacteria* (Fig. 6d; “A < F”). Many R- and F-associated bacteria were notably located close to the center of the ecosystem network (Supplementary Fig. S7), implying their importance in establishing the bacterial community network via plant-bacterium interaction. The *mle1-27* of the phylum *Myxococcota* as a PC3-contributor and *Sphingobium* of the phylum *Proteobacteria* as a network hub were notable taxa.

## Discussion

Accumulating evidence emphasizes the importance of microbiomes in controlling crop productivity that is a consequence of complex associations among microbiomes, plants, and abiotic factors. We investigated these associations in a unique upland fertilizer test field that has been stably managed for over 30 years. This allowed the generation of datasets of bacterial abundance and chemical components in soil. Soil properties clearly differed among the six test plots according to fertilizer protocols. Phosphate increased buckwheat growth (Fig. 1c,e), which confirmed previous results derived from the same field between 1992 and 1999^10^. Bacterial communities that were altered by crop cultivation periods became apparent. These findings revealed bacterial communities that participate in upland soil fertility and crop nutrition.

Hierarchical clustering sorted soil bacteria into three clusters in the manner dependent on nitrogen fertilizer and compost (Fig. 4). Three groups determined by co-occurrence network analyses largely overlapped with the clusters (Fig. 5). Composting was associated with more diverse microbial taxa than other soils (Table 1, Fig. 3d) and included 101 taxa that were specific to test plot C (Fig. 3f). The frequency of the phylum *Gemmatimonadota* was high in plot C soil (Fig. 3e, top) and that of taxa identified in this phylum belonged to cluster 3 (Supplementary Table S4). The paucity of taxa in plots NK, NP, and NPK suggested that nitrogen fertilizer affects bacterial diversity (Fig. 3d,f). The bacterial community structure correlates with inorganic nitrogen and organic fertilizers in other fields^8,18^. Taxa in the phylum *Actinobacteriota* were more diverse in cluster 2 than in the other clusters (Fig. 3f). Five of eight predominant taxa in cluster 2 belonged to *Actinobacteriota* (Supplementary Table S4), indicating that *these* bacteria dominated soil without either nitrogen fertilizer or compost. Taxa in the phylum *Chloroflexi* were more prevalent in cluster 1 (Fig. 3f and Supplementary Table S4), indicating that *Chloroflexi* dominated when soil was fertilized with nitrogen. Bacterial network hubs connecting the intra-and inter-groups were shared in all groups with *Acidobacteriota* and *Proteobacteria*, and specifically in groups 0, 1, and 2 with *Actinobacteriota* and *Chloroflexi* (Fig. 5d and Supplementary Table S6). These phyla were diverse and ubiquitous not only in soils from the test plots (Fig. 3f) but also in other types of soils^7^. Consequently, nitrogen fertilizer and compost were major determinants of the bacterial community structure in soils from the six test plots.

This study revealed linkage between bacterial community structure and pH in PCA and co-occurrence networks. The significance of pH to the soil bacterial community structure in the test plot soils indicates that it is an important predictor of bacterial community structure^19,20^. Our finding of lower soil pH in the nitrogen-supplemented NK, NP, and NPK plots is consistent with that fact that nitrogen fertilizer decreases the pH of various types of soils^11^. The results of the clustering analysis indicated that bacterial communities differed between soils fertilized with and without nitrogen fertilizer (Fig. 4h and Supplementary Table S3). This indicates that the link between pH and soil bacterial community structures is a consequence of nitrogen fertilization. Buckwheat tolerates acidic soils (pH 5.5‒6.0), in which plant biomass and seed yield increase^21,22^. The increased plant biomass might be linked to bacterial community structures in acidic soil. However, further investigation is required to identify specific bacteria that are associated with soil pH.

Topological analysis located group 0 at the center of the co-occurrence network (Fig. 5). The betweenness centrality parameter identified 25 nodes as intergroup or intragroup hubs in the network. The sole hub of chemical components was SO_4_^2−^ (Fig. 5c,d; “M”), which is a counter ion of the nitrogen fertilizer (NH_4_)_2_SO_4_. This is consistent with the indirect function of SO_4_^2−^ as a consequence of nitrogen/sulfate fertilizers. Sulfates derived from inorganic nitrogen fertilizers might have lowered the soil pH in the test plots. By contrast, SO_4_^2−^ can specifically maintain soil ecosystems through microbial activities that immobilize it to organic matter^23^. Such immobilization prevented an immediate increase in soil SO_4_^2−^ levels after nitrogen application and this induced them to increase later at the F stage (Figs. 2a, 6d and Supplementary Fig. S3). This agrees with the findings that the NP and NPK plots supplemented with nitrogen accumulated high levels of TOCs and changed the SO_4_^2−^ contents more dynamically.

The stage-dependent difference during cultivation revealed nine bacteria that were prevalent at the ripening stage (Fig. 6d). The frequencies of only three bacteria increased at the flowering stage, suggesting a unique plant-bacteria interaction in soils where buckwheat ripens. Two bacteria associated with ripening belonged to the large *Comamonadaceae* and *Burkholderiaceae* families. The order *Burkholderiales* is a dominant component of many soil ecosystems and includes species that promote plant growth, are endophytic^24^, and solubilize phosphate to a plant-available form^25^. These mechanisms potentially explain the association between ripening buckwheat and bacteria. This notion has attracted much attention in terms of understanding soil ecosystems in the field, especially in test plots consisting of andisols with an extremely high capacity to adsorb phosphoric acid. Another ripening-associated bacterium belongs to the genus *Nitrospira* that includes nitrogen dissimilatory and nitrite-oxidizing bacteria, as well as complete ammonia oxidizers (comammox)^26^, suggesting a relationship with plants via nitrogen dynamics in soil. The soils in the six test plots should be active in terms of nitrification because they accumulated little ammonium even on the second day of (NH_4_)_2_SO_4_ application (Supplementary Fig. S3). These results suggested a correlation between nitrifying bacteria and ripening buckwheat. However, their direct role in ripening awaits further investigation because nitrification is the result of complex interactions among available ammonium, plant root exudates, soil properties, comammox, and archaea^2^.

This study determined the impact of fertilizers on plant growth, soil bacterial community structure and chemical properties. Long-term fertilizer programs differentiated soil properties in the six test plots, generated bacteria that are key for a community structure, and promoted their temporal interactions with plants. A pioneer study in an open field system in Rothamsted, UK started in 1843 to investigate wheat production with rotations of potato, oats, beans, and other crops^9,27^. Buckwheat, rye, sweet potato, ground nuts, wheat and potato are uniquely rotated in the test field assessed herein. Among these crops, we focused on buckwheat, of which 1.6 million tons were produced globally during 2019 (FAOSTAT)^28^. Our findings will guide the development of future agricultural technology to control bacterial community structures and improve buckwheat productivity. We plan to investigate ecosystems in the test plots rotating the six crops. The data should reveal unique ecosystems associated with stably managed crop rotations and facilitate the development of practical strategies to design and control bacterial community structures and plant cultivation.

## Materials and methods

### Fertilizer test field and soil sampling

The fertilizer test field is located at T-PIRC, University of Tsukuba, Japan (36° 07′ 07″ N, 140° 05′ 44″ E) (https://farm.t-pirc.tsukuba.ac.jp/en/), where the climate is classified by Köppen–Geiger as temperate. The soil type is an andosol^29^ derived from volcanic tephra and accumulated humus^29^. The field is separated into 12 plots (10 × 4 m each) that have been managed under continuous fertilizer programs since 1986 (Fig. 1)^10^. Ground nut, fallow, potato, buckwheat, rye, sweet potato, wheat, buckwheat, and rye are rotated every four years (Fig. 1b). The six plots used in this study were fertilized one day before sowing without (plot 0), or with potassium chloride and calcium dihydrogen phosphate (KP), ammonium sulfate (N) and potassium chloride (NK), ammonium sulfate and calcium dihydrogen phosphate (NP), three macronutrients (NPK), and rice straw compost matured with water for ~ 2 years (C). The plots were treated with 1.5 g/m^2^ each of chemical fertilizers and compost. Buckwheat (*Fagopyrum esculentum* Moench 'Hitachiakisoba') was cultivated with a ridge width of 30 cm at a sowing density of 5 g/m^2^. Buckwheat seeds were purchased from (Public Interest Incorporated Association) Ibaraki Prefectural Agriculture and Forestry Promotion Corporation (Mito, Japan). Soils in the plots were collected at the following stages throughout buckwheat cultivation: before fertilization (B, − 11 days after sowing; DAS), after fertilization (A, 0 DAS), flowering (F, 23 DAS), ripening (R, 52 DAS), and post-harvest (H, 72 DAS) (Fig. 1d). Stage B corresponds to the interval between potato harvesting and buckwheat cultivation. Soil at stage A soil was collected on the day before sowing (0 DAS). Four batches of soil were simultaneously sampled once at a depth of 2–15 cm for the tillage layer from each test plot (Supplementary Fig. S1).The samples were passed through a 2-mm mesh, dispensed into ~ 1 g portions, and stored at − 80 °C. Residual bulk soils were stored at 10 °C. We analyzed the chemical components and amplicon sequences of the four batches sampled from each plot at each cultivation stage (*n* = 4 per plot).

### Measurement of soil chemical components

Soils (5 g) were dried at 120 °C for 15 min to determine the water content using an MOC63c moisture analyzer (Shimadzu Co., Kyoto, Japan) as described by the manufacturer. Plant materials and stones were removed, then soils (6 g wet weight) suspended in 30 mL of H_2_O were reciprocally shaken at 160 rpm for 1 h at room temperature. The supernatant (15 mL) was obtained after solid matter settled for 1 h. The pH was measured in 5-mL portions. Aliquots (4 mL) were filtered through a 0.45-µm porous membrane (Millex HP, Merck KGaA, Darmstadt, Germany). Thereafter, Na^+^, sK^+^, NH_4_^+^, sCa^2+^, sMg^2+^, Cl^−^, NO_3_^−^, and SO_4_^2−^ were compared with Multication and Multianion Standard Solutions III (Fujifilm Wako Pure Chemical Co., Osaka, Japan) using an HIC-20A Super ion chromatograph (Shimadzu Co.).

Soils (1.5 g) were suspended in 30 mL of Component Extracting Solution (pH 3.4) (EW-T201J, Air Water Inc., Osaka, Japan), vortex-mixed for 3 min, then reciprocally shaken for 20 min. After settling for 7 min, supernatants were passed through a GA-55, glass fiber filter (Toyo Roshi Kaisha, Ltd., Tokyo, Japan). Insoluble materials in the filtrate were removed by passage through a 0.45-µm porous membrane if necessary. Levels of aPO_4_^3−^, eK^+^, eCa^2+^, and eMg^2+^ were colorimetrically determined using EW-THA1J and EW-T102J soil analyzers as described by the manufacturer (Air Water Inc.). Soil carbon contents were quantified using a TOC-L CPN analyzer equipped with an SSM-5000 solid sample module (Shimadzu Co.). Soil samples (0.3 g) were dispensed onto a sample boat (638-92099, Shimadzu Co.) and combusted at 900 °C. Total carbon (TC) was monitored to determine the amount of CO_2_ produced. The amount of generated CO_2_ was also quantified to determine inorganic carbon (IC) by heating 0.3 g of soils in phosphoric acid at 200 °C. The TOC was calculated by subtracting IC from TC.

### Crop and meteorological data collection

Twenty individual plants were collected from each plot. Shoot height was measured on 23 and 71 DAS. Yields of stems, leaves, and seeds in two areas 3.6 m^2^ each (4 rows × 3 m) in each plot were measured. Seed weight (yield) and biomass of stems and leaves were measured after harvest. A HOBO U30-NRC weather station (Onset Computer Co., Bourne, MA, USA) was installed near the fertilizer test field and activated on 26th August 2019 to measure air, ground temperatures at a depth of 15 cm, humidity, solar radiation, photosynthetically active radiation, rainfall, wind speed, and wind direction every 5 min. Graphs of these parameters were created using Excel for Microsoft 365 MSO v. 2112 (Microsoft Corp., Redmond, WA, USA).

### Sequencing 16S rRNA gene amplicons

We extracted DNA from soil using DNeasy Power Soil Pro Kits (Qiagen Sciences Co., Hilden, Germany) as described by the manufacturer except for adding 40 mg/g soil of skim milk (Nakalai Tesque Inc., Kyoto, Japan) to the extraction buffer. An amplicon library of the V1–V2 region of the 16S rRNA gene was constructed using the (5′ → 3′) primer set^30^: 16S_27Fmod: TCGTCGGCAGCGTCAGATGTGTATAAGAGACAGAGRGTTTGATYMTGGCTCAG

and 16S_338R: GTCTCGTGGGCTCGGAGATGTGTATAAGAGACAGTGCTGCCTCCCGTAGGAGT. Nucleotides were pair-end sequenced using the MiSeq system (Illumina Inc., San Diego, CA, USA). Raw sequencing reads were processed and low-quality reads were eliminated using Qiime2 v. 2020.11. Amplicon sequence variants (ASVs) were produced using DADA2^31^ and a naïve Bayesian classifier^32^, then their taxonomies were assigned using SILVA v.138.1^33,34^.

### Dataset construction

Supplementary Table S1 shows the original amounts of chemical components and bacterial ASVs in four soil batches per plot and cultivation stage. No data are available for soil sample 28 because the abundance of extracted DNA was insufficient to analyze sequences. Supplementary Dataset 1 lists the median values of each of the four batches shown in Supplementary Table S1, and eliminated 753 taxa missing at all cultivation stages in all test plots. Values for the relative frequency of the remaining 870 taxa and 14 chemical components were standardized using the scale function of R v. 3.6.2^35^ to obtain Supplementary Dataset 2. Statistics were analyzed and graphs were prepared using both Supplementary Datasets.

### Statistical analysis of soil chemicals

A heatmap of soil chemical components was prepared using Supplementary Dataset 2 and the heatmap.2 function in the gplots package^36^ in R to visualize changes that depended on each plot and cultivation stage. Principal components were analyzed using the prcomp function in R and chemical component data in Supplementary Dataset 2. Significance was tested between two soil groups using permutational multivariate analysis of variance (PERMANOVA). Ratios of standard deviation to average (CV) (Supplementary Fig. S1) were calculated from the data in Supplementary Table S1. Soil chemical properties were visualized (Supplementary Fig. S3) using the boxplot function in R and data in Supplementary Table S1.

### Bacterial community analysis

Box plots were created based on the numbers of 16S rRNA gene amplicon reads (Supplementary Table S1) and taxa in Supplementary Dataset 1 in R and visualized using the barplot function in R. Supplementary Dataset 1 was used to determine the relative frequency of taxa that appeared in the soil samples. The numbers of taxa within each phylum were counted with reference to Supplementary Dataset 1. We determined the Shannon index^37^ of soil bacterial communities in each test plot per cultivation stage using rarefied data from Supplementary Dataset 1.

### Analysis of correlations between bacterial communities and chemical components

Principle components were analyzed using the prcomp function in R and bacterial community data in Supplementary Dataset 2. Ordination vectors of the chemical components determined using envfit function in the vegan package^38^ and the chemical component information in Supplementary Dataset 2 that correlated with variations of bacterial community (*p* < 0.01) were projected onto the PC1–PC2 scatter plot. Correlations between bacterial taxa and PC3 scores were evaluated as load quantity (LQ3) defined as:

$${LQ3}_{i}=\sqrt{{l}_{3}}\times {h}_{3i},$$

where *i* is taxon, *l*_*3*_ is variation (eigenvalue) of third principle component, *h*_*3i*_ is the third component of eigenvector of taxon *i*. Load quantity 3 was obtained using the sweep function, and standardized using the scale function in R (z-LQ3). Heatmaps and dendrograms of bacterial community (Fig. 4h) were created by complete linkage using Supplementary Dataset 2 and the heatmap.2 function in the gplots package and the dist and hclust functions in R. Taxa were sorted into three clusters using the cutree function (k = 3) in R. A Venn diagram was created using a list of taxa correlating with chemical components (absolute value of *r* was higher than 0.6) and the venn function.

### Co-occurrence network analysis

Correlation coefficients (*r*) among data in Supplementary Dataset 2 were determined using the cor function in R. A data frame for co-occurrence network analysis was created using the reshape2^39^ and igraph^40^ packages in R. Briefly, the *r* matrix table was converted to a one-by-one list and paired with *r* categorized to the redundant half (upper right). Coefficients that correlated with themselves (on the diagonal) were discarded, then pairs with absolute *r* values of > 0.6 were extracted. The resulting list was formatted using the graph.data.frame function. Co-occurrence networks were visualized based on the Kamada–Kawai algorithm^41^ using the plot function in the igraph package.

The largest complete graph in the co-occurrence network was uncovered using the cor, melt, graph.data.frame functions and the igraph package in R, Excel, and Supplementary Dataset 2. The *n* nodes with the most edges were extracted from nodes of the bacteria and the chemical components arranged in descending order of the number of edges, and when the *n*th node had *n* − 1 or more edges. This operation was repeated until all extracted nodes had *n *− 1 edges and to uncover the center of the groups.

The numbers of edges (degree centrality) and betweenness centrality^13^ of individual nodes were respectively determined using the degree and betweenness functions in R. Within-group degrees (numbers of edges that linked nodes in the same group) and participation coefficients were calculated to characterize nodes as hubs in the network using Excel^14–16^. Participation coefficients of the nodes were defined as described^14^. Taxa with standardized within-group degrees of > 1.96 were defined as the intragroup hub. Taxa with a participation coefficient > 0.5 were defined as the intergroup hub. These hubs were extracted from Supplementary Dataset 2 and their linkages were visualized using the plot function as described above before being arranged to a schematic graph.

### Temporal changes in bacterial communities

Differences in the relative frequency of taxa between a cultivation stage and its preceding cultivation stage (Δf) is defined as:

$${\Delta f}_{xpn}={f}_{xpn}-{f}_{xp\left(n-1\right)},$$

where *f, x, p, n* are relative frequency, taxon, test plot, and cultivation stage, respectively. The Δf values in each test plot and cultivation stage were plotted using the beeswarm function, the principal components were analyzed using the prcomp function in R. Taxa with a positive Δf in all plots at any cultivation stage (*p* < 0.05, *t*-test) were selected. The relative frequency of resultant taxa was visualized in a heatmap together with fluctuating chemical components (SO_4_^2−^, NO_3_^−^, sCa^2+^, sMg^2+^) and the height of buckwheat was determined using the heatmap.2 function in R.

### Statistical tests

All data were statistically analyzed using R. Homoscedasticity between groups was assessed by F-test using the var.test function in R. Paired and three or more groups were compared using Student *t*-test (t.test function) and Tukey–Kramer (aov and TukeyHSD functions) test, respectively. The significance of the PCA findings was tested by the Euclidean method using adonis2 (PERMANOVA). The significance of correlations was tested using the cor.test function.

### Ethics statement

Experimental research and field studies on buckwheat, including the collection of buckwheat comply with relevant institutional, national, and international guidelines and legislation.

### Supplementary Information

Dataset S1.

Dataset S2.

Supplementary Figures.

Supplementary Table S1.

Supplementary Table S2.

Supplementary Table S3.

Supplementary Table S4.

Supplementary Table S5.

Supplementary Table S6.

## Data Availability

All data supporting the conclusions of this research are provided in this article and in supplemental files. The data of the 16S rRNA gene sequencing have been deposited with links to BioProject accession number PRJDB15840 in the DDBJ BioProject database. This paper does not report original code.
