# Supplementary material for: Temporal and fertilizer-dependent dynamics of soil bacterial communities in buckwheat fields under long-term management
Source: Sci Rep. 2024 Apr 30;14:9896. doi: 10.1038/s41598-024-60655-w (PMC11061196; doi:10.1038/s41598-024-60655-w)
Supplement: Supplementary file 3 — Supplementary Figures. [file 41598_2024_60655_MOESM3_ESM.pdf]

Temporal and fertilizer-dependent dynamics of soil bacterial communities in buckwheat fields under long-term management

Susumu Morigasaki, Motomu Matsui, Iwao Ohtsu, Yuki Doi, Yusuke Kawano, Ryosuke Nakai, Wataru Iwasaki, Hisayoshi Hayashi, Naoki Takaya

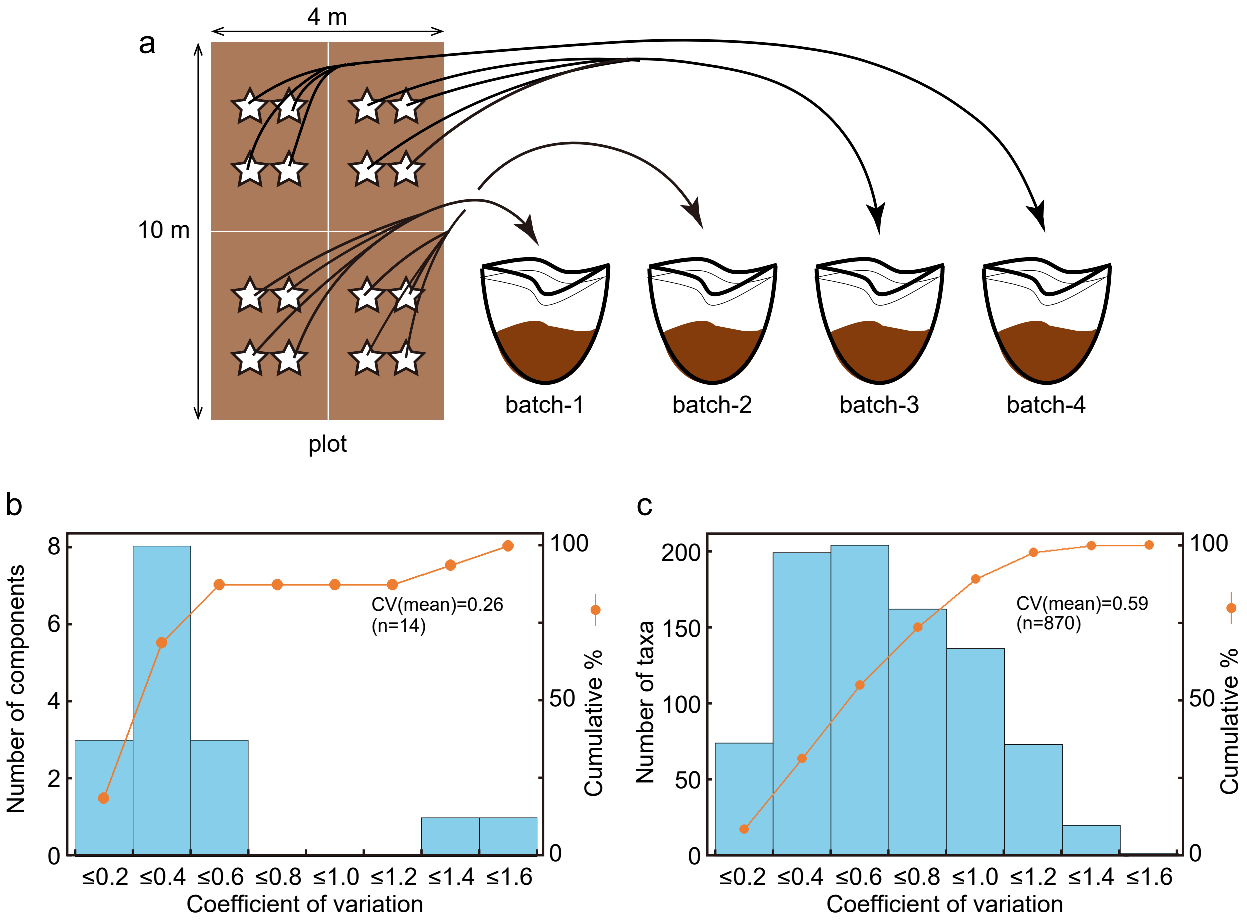

Figure S1. Soil sampling method and variation of data

(a) Batches comprise mixtures of four samples from different sites in one quarter of each plot (unfilled stars; four batches per plot. **(b and c)** Coefficient of variation (CV) (= SD / mean) was determined using data of four batches from a test plot at one sampling using data in Table S1. Mean of 30 CV values (6 plots × 5 samplings) for each component or taxon was subjected to drawing frequency distribution (histogram, left scale) and their cumulative ratio (%) (orange line, right scale) by the hist, cut, and numeric functions in R. **(b)** Chemical components. Insert is mean of CVs except aPO_4^{3-} (CV = 1.4) and NH_4^+ (CV = 1.5). **(c)** Bacterial abundance. Insert is mean of CVs of 870 taxa.

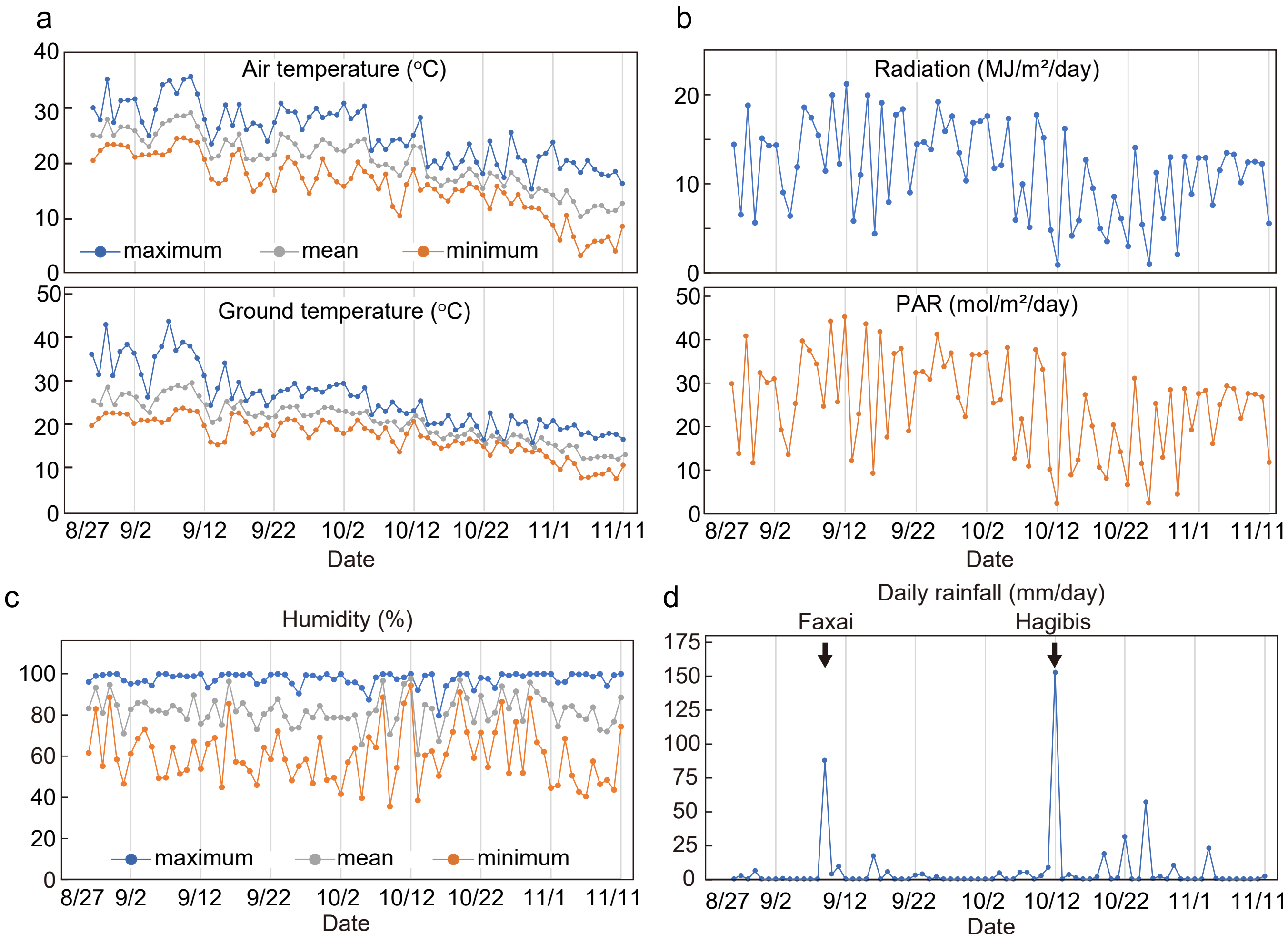

Figure S2. Meteorological information

(a) Maximal, minimal and means of daily temperatures in air and 15 cm below ground. **(b)** Daily solar and photosynthetically active radiation (PAR). **(c)** Maximal, minimal and means of relative humidity. **(d)** Daily rainfall (precipitation). Arrows, 9th September and 12th October days when typhoons Faxai and Hagibis respectively hit Tsukuba.

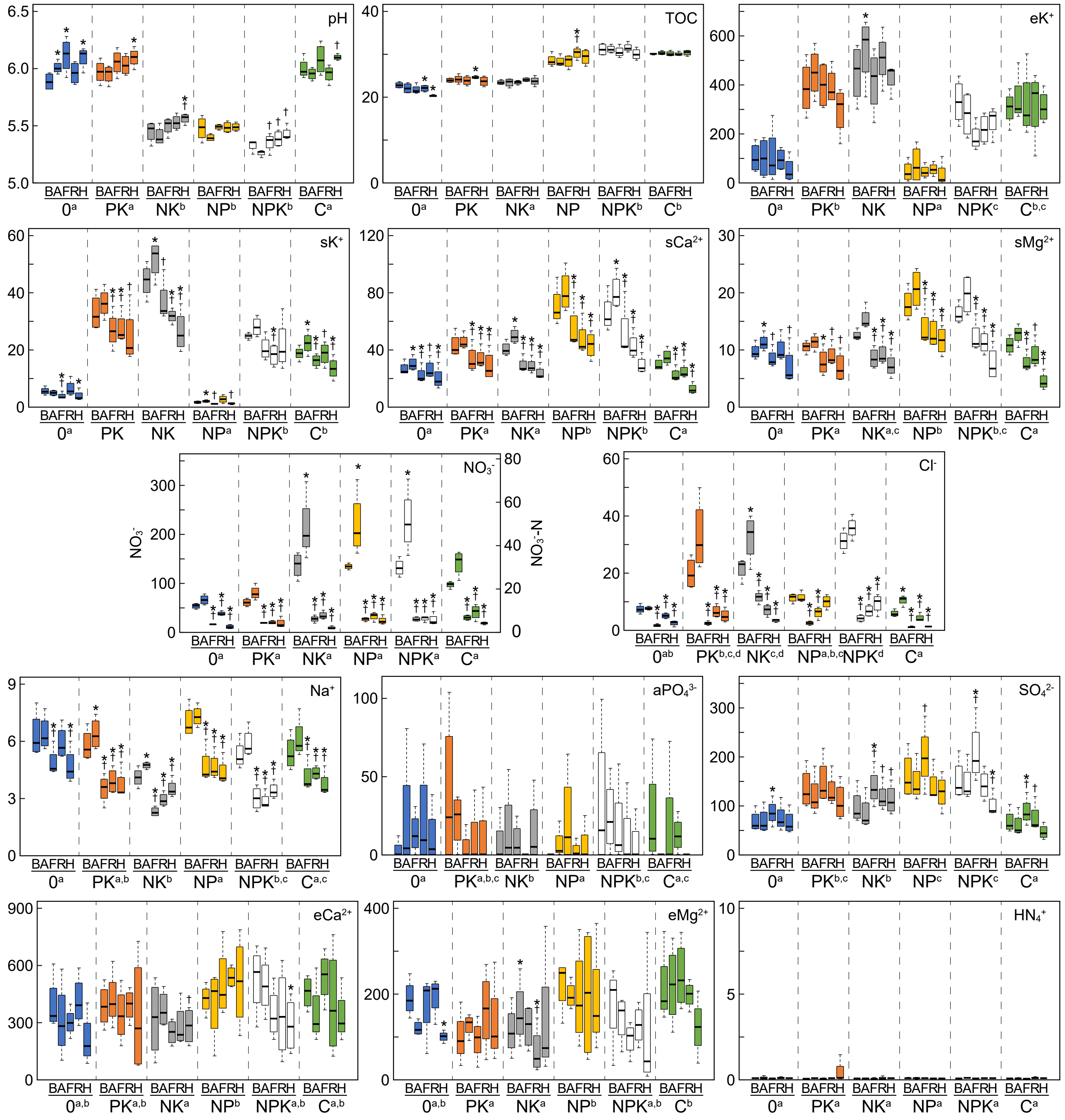

Figure S3. Features of soil chemical components that are dependent on cultivation stage and fertilizer

Box plots show chemical components in soils sampled before (B) and after (A) fertilization, flowering (F), ripening (R) and harvest (H) stages (n = 4). A unit of vertical axis is mg / kg dry soil, except for TOC (g / kg dry soil) and pH. NO_3^--N is calcutlated by an equation: NO_3^--N = NO_3^- × 0.2259. Thick line in box, median; top of box, third quartile (Q3); bottom of box, first quartile (Q1); upper whisker, maximum value (< Q3 + 1.5 × [Q3 – Q1]); lower whisker, minimum value (> Q1 - 1.5 × [Q3 – Q1]). Test plots are shown as 0, blue; PK, orange; NK, grey; NP, yellow; NPK, white; C, green.

* Significant difference from stage B (p-value < 0.05, t-test). † Significant difference from stage A (p-value < 0.05, t-test).

Test plot names with the same superscript letters do not significantly differ (p ≥ 0.05, Tukey-Kramer test).

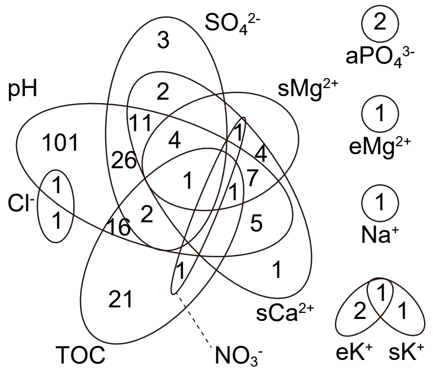

Figure S4. Number of taxa correlated with chemical components

Venn diagram shows numbers of taxa that correlated with chemical components (|r| > 0.6). Bacterial taxa that were correlated with chemical components (|r| >0.6) were listed. Using the list, the venn function created a draft diagram with number of bacterial taxa correlated with pH, TOC, SO_4^{2-}, sCa^{2+}, sMg^{2+} in R. The Venn diagram was completed by manually adding results on the other chemical components.

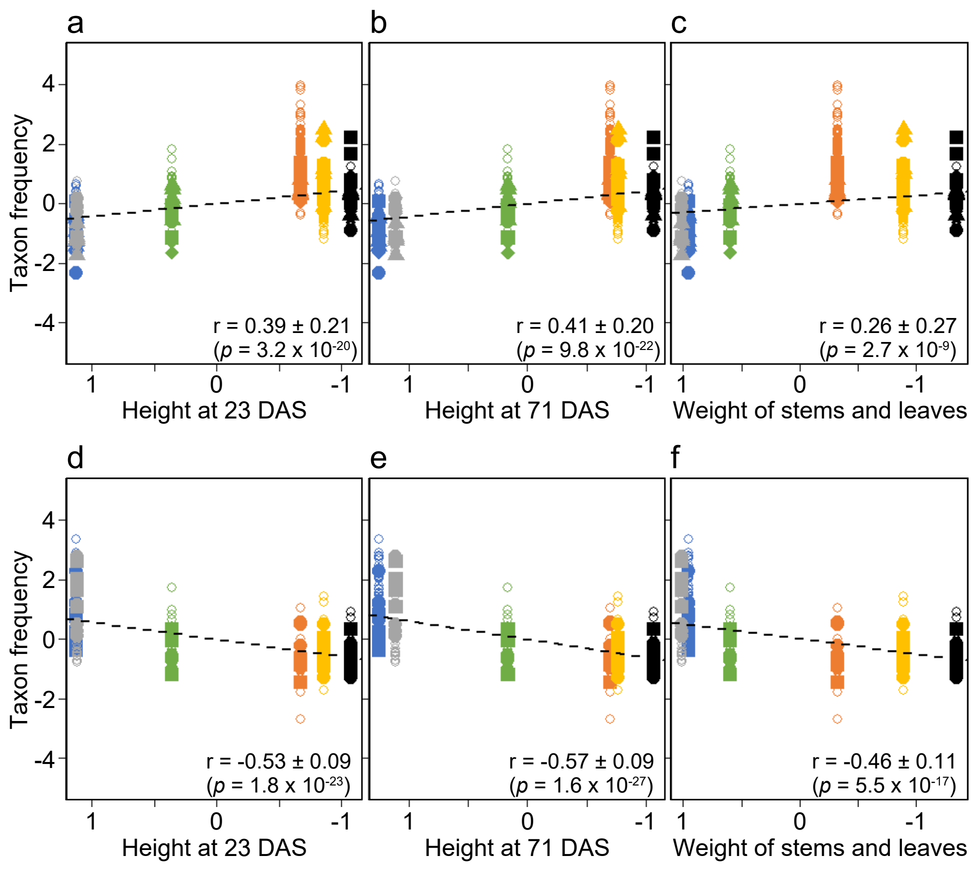

Figure S5. Correlation between buckwheat traits and frequency of bacteria with extramely high/low PC3 load quantity

Frequencies of taxons listed in Table S4 and buckwheat traits for height and weight of terrestrial parts were standardized. Blue, plot 0; orange, plot PK; grey, plot NK; yellow, plot NP; black, plot NPK; and green, plot C. Correlation coefficient (r) was indicated inside each panel (mean ± SD). **(a–c)** Correlation of frequencies of taxa having standardized PC3 load quantities (LQ3s) higer than 2.33 to buckwheat traits: **(a)** height at 23 DAS, **(b)** height at 71 DAS, and **(c)** weight of stems and leaves at 71 DAS. Closed symbols indicate four taxa with highest r : (square) *Actinobacteriota; Actinobacteria; Corynebacteriales; Nocardiaceae; Rhodococcus;_*, (circle) *Proteobacteria; Gammaproteobacteria; CCD24; CCD24; CCD24; metagenome*, (triangle) *Actinobacteriota; Thermoleophilia; Gaiellales; uncultured; uncultured;_*, (diamond) *Proteobacteria; Gammaproteobacteria; Diplorickettsiales; Diplorickettsiaceae; Diplorickettsiaceae; uncultured_gamma*. Open circles indicate the other 13 taxa. **(d–f)** Correlation of frequencies of taxa having standardized PC3-LQ lower than -2.33 to buckwheat traits: **(d)** height at 23 DAS, **(e)** height at 71 DAS, and **(f)** weight of stems and leaves at 71 DAS. Closed symbols indicate two taxa with lowest r : (square) *Myxococcota; Polyangia; mle1-27; mle1-27; mle1-27; uncultured_proteobacterium* and (circle) *Chloroflexi; P2-11E; P2-11E; P2-11E; P2-11E; uncultured_bacterium*. Open circles indicate the other 8 taxa.

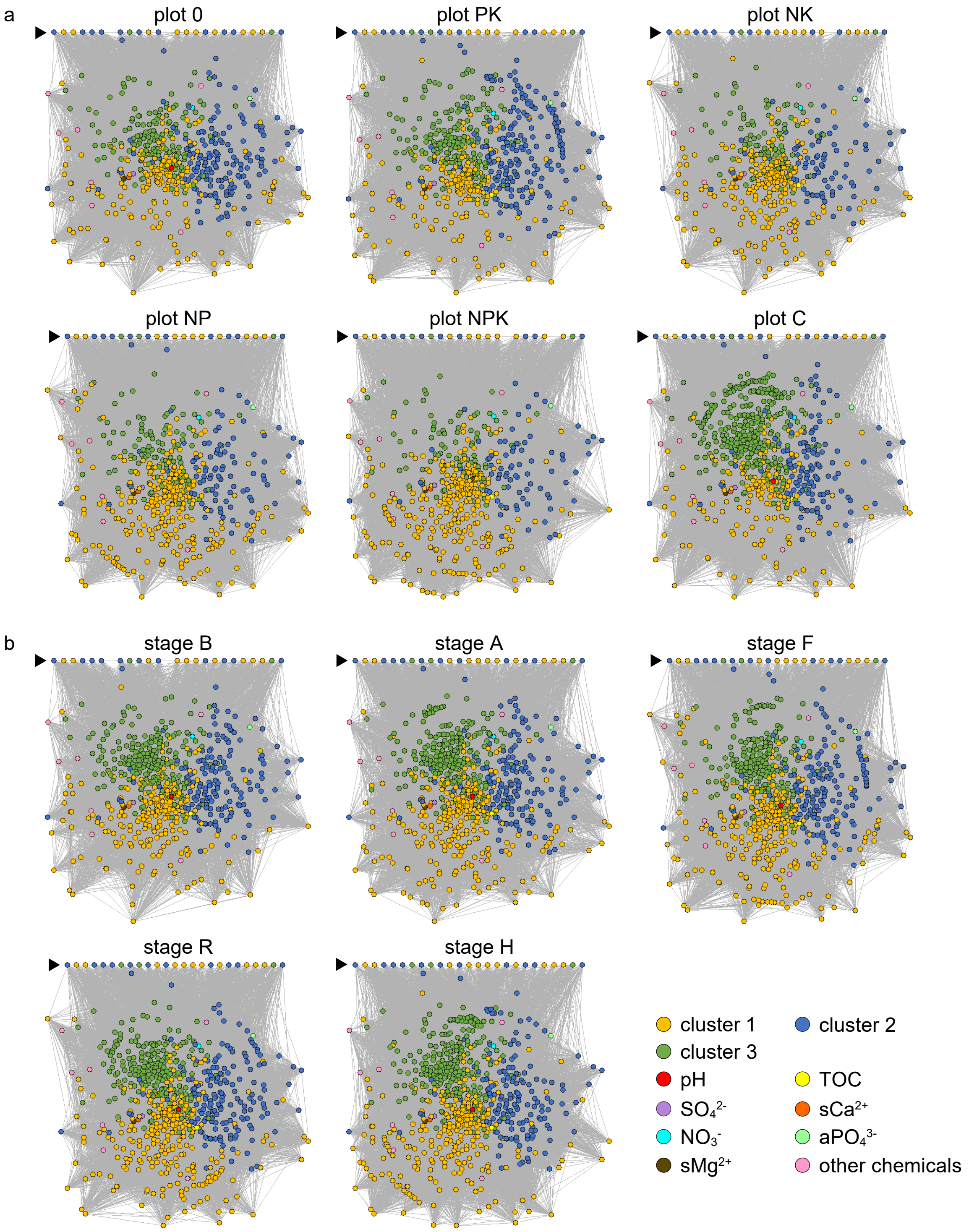

c

plot	total number		degree centrality			betweenness centrality			closeness centrality			path length		
	nodes	edges	mean	SD	* TK test	mean	SD	* TK test	mean	SD	† TK test	mean	SD	* TK test
6 test plots	859	22928	53	54		912	1195		0.000388	0.000075		3.126	1.118	
0	515	36755	143	21	a	186	110	a	0.001130	0.000027		1.722	0.454	b, c
PK	573	45891	160	25		206	113	b	0.001017	0.000026		1.720	0.454	a, c
NK	432	26272	122	19		155	89	c	0.001352	0.000035		1.718	0.457	a
NP	505	35756	142	28	a	181	129	a	0.001155	0.000037		1.719	0.456	a, c
NPK	473	30916	131	22		171	127	a, c	0.001231	0.000034		1.723	0.454	b, c
C	608	51082	168	27		219	135	b	0.000957	0.000025		1.723	0.452	b

stage	total number		degree centrality			betweenness centrality			closeness centrality			path length		
	nodes	edges	mean	SD	* TK test	mean	SD	* TK test	mean	SD	* TK test	mean	SD	* TK test
B	615	48813	159	64		230	261	a	0.000935	0.000060		1.748	0.455	
A	669	61857	185	78	a	243	260	a	0.000871	0.000062	a	1.727	0.458	
F	670	61149	183	74	a	247	283	a	0.000864	0.000062	a, b	1.739	0.470	a
R	674	59677	177	71	a	249	261	a	0.000857	0.000055	b	1.741	0.452	a, b
H	682	61185	179	74	a	253	341	a	0.000847	0.000055		1.742	0.454	b

Figure S6. Co-occurrence network in test plots and in cultivation stages

Data of bacterial community and chemical properties are divided **(a)** per test plot or **(b)** per cultivation stage.

(a and b) Coordinates (layout) of nodes are same as Fig. 5a, except that taxon nodes lost in Fig. 5a are placed at the top of panels (closed arrowhead). Node color indicates bacterium cluster and chemical properties, as shown in right bottom in (b).

(c) Topological property parameters of networks. Degree, betweenness, and closeness centralities and average path length are determined using degree, betweenness, closeness, and distance functions in R. * Means with the same letter are not significantly different (p > 0.05, Tukey-Kramer test). † P-values between any plots are less than 0.05 (Tukey-Kramer test).

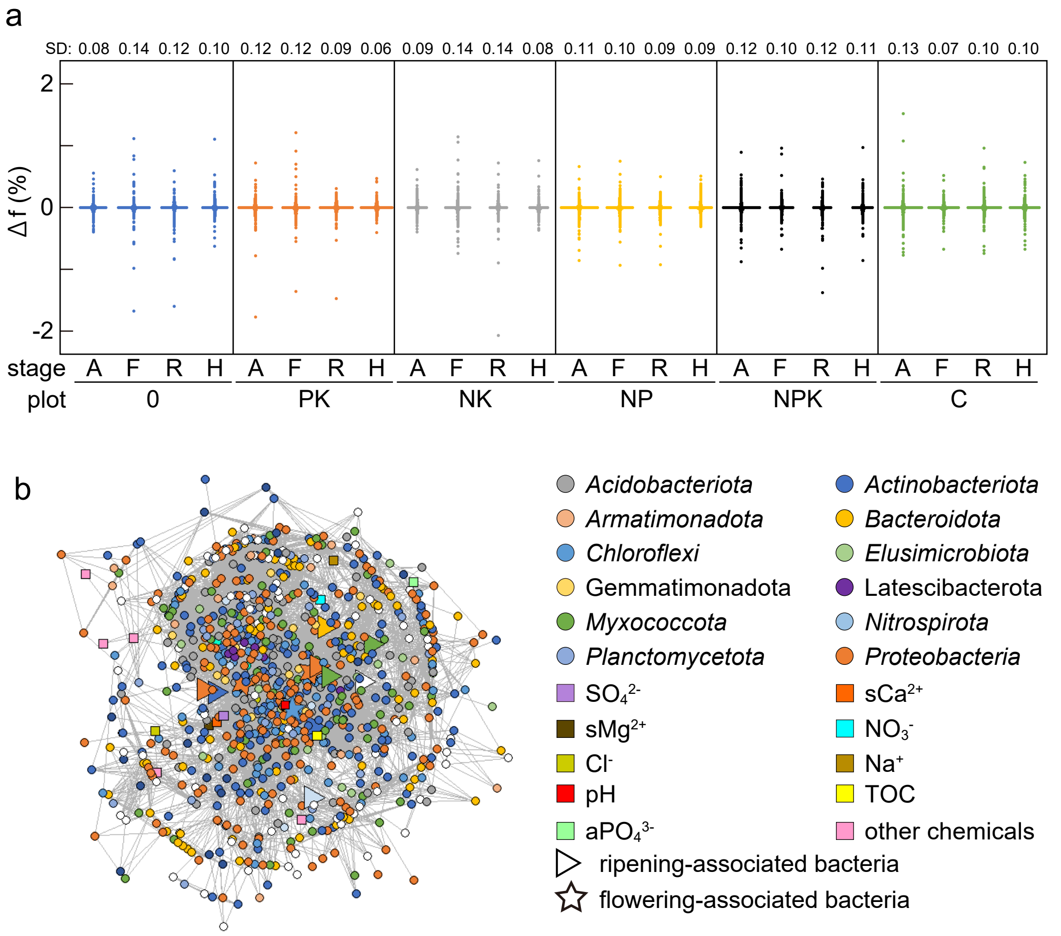

Figure S7. Temporal change of relative frequency of bacterial taxa

(a) Beeswarm plots of differences in relative frequency calculated using equation Δf(n)=f(n)-f(n-1), where f is frequency, Δf is difference in frequency, n, given cultivation stage; n-1, stage before given stage. Significance was tested by Tukey-Kramer test, resulting in no significance. **(b)** Network indicating the locations of bacterial taxa associated with buckwheat (triangles and stars). Node color represents belonging phylum. Square nodes are chemical components.
